# Book Reviews

**Published:** 1885-11

**Authors:** 


### BOOK REVIEWS.

OHRENHEILKUNDE. *von* DR. A. SARROU.

The writer's very natural object is to impress the general practitioner with the importance of his subject. To this end he speaks of the frequency of ear trouble. He mentions an examination of 6,000 school children, in whom inspissated cerumen, a retraction, or calcareous infiltration of the drum-head, was found in 22 per cent. of the boys, and 23 per cent. of the girls. He further quotes Schwartze as finding, in autopsy, a creamy or green-yellow fluid in the labyrinth, tympanum, etc., in 40 per cent. of all the children examined. He emphasizes the slightness of the barriers everywhere existing between the ear and the interior of the skull. There are enough possible disastrous results, he thinks, from a suppurative otitis externa, to bring otorrhœa from the province of the specialist into that of the general practitioner. He alludes to a class of cases where an otorrhœa, causing only moderate deafness, is allowed to run on for years, until finally it developes a brain-trouble, and the patient dies suddenly of "Heart Disease," or some other covenient diagnostic scape-goat. He quotes Wilde: "So long as otorrhœa is present, we can never tell how, when, or where it will end."

Some of his suggestions on diagnosis are very good. He warns the physician against setting down as Menière's disease every ear trouble with vertigo. He thinks this symptom is possible in any pathological condition of the ear. He calls attention to the fact that the muscles of the tube regulate the ventilation of the tympanum, and that, through general debility, or exhaustion from any cause, there may result, from the inactivity of these muscles, a closure of the Eustachian tube with tympanic catarrh, retraction of the drum-head, and a considerable degree of deafness,—while the catheter finds the tube pervious, and, apparently, in a normal condition. The diagnosis of many a vague case of " rheumatism " of the neck, with occasional fever and peculiar pains extending towards the occiput, might, he thinks, be cleared up, if the physician remembered the possibility of caries of the bones of the ear. He mentions the striking similarity between the symptoms of phlebitis with the etiology of suppurative otitis, and those of a beginning typhoid fever. Under the head of thrombosis, he speaks of one symptom which he has never seen described, but which, he believes, is seldom lacking, viz., the bronze discoloration of the skin,—which symptom he has seen twice lead to a wrong diagnosis of morbus Adissonii. The conjunctiva was normal. The methods he recommends for localizing the cause of deafness are especially good. He emphasizes the value of the large tuning fork, with Politzer's modifications, by which the over-tones are avoided, and the scale can be raised or lowered at will. By this means it can be determined whether the difficulty in hearing is in the high or low notes. He quotes Moos, who holds that in chronic middle-ear catarrh, the power to hear high notes is impaired; while in affections of the special nerve-apparatus, the perception of the middle notes is disturbed. If the higher of these middle notes cannot be heard, the lower spiral is diseased; otherwise, the upper spiral.

In treatment, Dr. Sarrou's governing principle seems to be, as in one place he quotes, " Primum est non nocere." As an air-douche, he prefers Politzer's bag. He describes a complicated apparatus, somewhat resembling Waldenburg's compressed air cylinders, for equalizing and increasing the pressure of the air; "but," he remarks, "while such an apparatus would form an imposing addition in a consultation-room, too great pressure has cost more than one patient his life, and here, also, the old proverb holds true,—that the most skillful physician finds his best instrument in an intelligently guided hand." He denounces Valsalva's method, especially where there is an atheromatous condition of the arteries, as causing an intense hyperæmia of the whole head. In this connection he gives a practical suggestion on the right use of the handkerchief. He considers "Schneützen à la paysan"—if it be allowed to so mingle languages,—as the truly physiological way; *i. e.*, under cover of the handkerchief, closing each nostril in turn, and by a forcible expiration, expelling the mucus from the free aperture. For suppurative otitis externa, he blows in dry boracic acid, and uses absorbing cotton, dry, instead of syringing. In middle-ear catarrh, he advises paracentisis, as soon as perforation threatens; or where this is impracticable, as in small children, he finds Politzer's air-douche often of great benefit. In scarlet fever and diphtheria, he keeps absorbent cotton, soaked in a 10 per cent. solution of salicylic acid, in the external meatus, as well as in the nose.

In cases of cicatricial contraction of the Eustachian tube, he describes a very delicate operation for keeping open the incision in the membrane. It consists in dissecting off a triangular piece, from the outer layer of the drum-head, and inserting this into the opening. However, as there is a large element of chance in this, he does not recommend it, but suggests the simple device, for counteracting the retraction of the membrane, of keeping oil-soaked absorbing cotton in the external meatus.

He mentions the troublesome *tinnitus aurium* resulting from the use of cocaine, in many cases, which fact, he thinks, will prevent its ever becoming very popular in the treatment of ear-diseases.

The writer makes no claim to originality, but simply desires his book to be of use. It seems probable that no physician will read it through without feeling that the writer's aim has been realized. H. H. C.

---

Applied Medical Chemistry. *By* Lawrence Wolff, m. d. *8vo., pp 174. Philadelphia:* P. Blakiston Son & Co.; *Chicago:* W. T. Keener.

It would be hard to tell what motives influenced the author in writing this book. In the preface it is stated that he wished to present certain matters in a simple and concise manner, but as the body of the work shows scarcely a trace of this *conciseness* or *simplicity*, one is inclined to think he must have forgotten this in following some other object. What this other object is, one fails to learn on a close examination.

A student beginning chemistry would certainly find great difficulty in understanding many of the author's explanations, as they are frequently made in most barbarous and unscientific English, and at times are even lacking in accuracy. As illustrations of obscure and careless English, the reader may refer to page 14, under the head of "Specific Gravity," to page 31, under "Stoichiometry," and to page 83, under "Alcohols." From the title page it appears that the author is a member of several learned societies and an instructor in an Eastern medical college. In addition to all this, it must now be said he has *written a book.* J. H. L.

A TEXT-BOOK OF MEDICAL CHEMISTRY. *By* E. H. BARTLEY, M. D. *12mo., pp. 376. Philadelphia:* P. BLAKISTON SON & CO.; *Chicago:* W. T. KEENER.

PROFESSOR BARTLEY, the well-known chemist of Brooklyn, has given us here an excellent work, accurate and well arranged.

He evidently appreciates the needs of medical students, and he has witten a text-book which deserves attention at the hands of teachers. There was a time when chemistry was a mere "side-show" in a medical college, and anything would do as a text-book; but modern methods are obtaining a hearing here as elsewhere, and the large number of medical chemistries afforded to students attest the efforts made in this direction. Professor Bartley's book is certainly one of the best of these. J. H. L.

